# Adoption of Task-Specific Sets of Visual Attention

**DOI:** 10.3389/fpsyg.2017.00687

**Published:** 2017-05-09

**Authors:** Mike Wendt, Svantje T. Kähler, Aquiles Luna-Rodriguez, Thomas Jacobsen

**Affiliations:** ^1^Faculty of Human Sciences, Medical School HamburgHamburg, Germany; ^2^Experimental Psychology Unit, Helmut Schmidt University/University of the Federal Armed Forces HamburgHamburg, Germany

**Keywords:** preparation, task switching, visual attention, response caution, executive functions

## Abstract

Evidence from behavioral and physiological studies suggests attentional weighting of stimulus information from different sources, according to task demands. We investigated the adoption of task-specific attentional sets by administering a flanker task, which required responding to a centrally presented letter while ignoring two adjacent letters, and a same-different judgment task, which required a homogenous/heterogeneous classification concerning the complete three-letter string. To assess the distribution of attentional weights across the letter locations we intermixed trials of a visual search task, in which a target stimulus occurred randomly in any of these locations. Search task reaction times displayed a stronger center-to periphery gradient, indicating focusing of visual attention on the central location, when the search task was intermixed into blocks of trials of the flanker task than into blocks of trials of the same-different task (Experiment 1) and when a cue indicated the likely occurrence of the flanker task as compared to the likely occurrence the same-different task (Experiment 2). These findings demonstrate flexible adoption of task-specific sets of visual attention that can be implemented during preparation. In addition, responses in the intermixed search task trials were faster and (marginally significantly) more error-prone after preparation for a (letter) task repetition than for a task switch, suggesting that response caution is reduced during preparation for a task repetition.

## Introduction

In task switching studies participants frequently alternate between different choice reaction time (RT) tasks afforded by the same stimuli. These studies have provided ample evidence that task performance, in particular in task switch trials (i.e., trials in which the to-be-executed task differs from the task of the directly preceding trial), benefits from increasing the length of a preparation interval during which participants have foreknowledge about the identity of the upcoming task (for overviews, see Karayanidis et al., 2010; Kiesel et al., 2010). This facilitation is usually attributed to *task-set preparation*, that is, to a set of processes that configure the cognitive system to a state that enhances speed and/or accuracy of processing an upcoming stimulus according to the requirements of the currently relevant task. Task preparation thus constitutes an important means to enhance processing efficiency, particularly in conditions of changing task requirements for which it cannot be assumed that a state of task-specific readiness is simply carried over from previous application.

Tasks typically used in task switching experiments, albeit cognitively simple, involve a variety of different components as possible candidates for preparatory optimization, and the precise set of components may depend on the specific combination of tasks between which switching is required. A frequent situation involves tasks associated with perceptually different target dimensions, such as shape and color. Task preparation in such a context may involve biasing attentional weights in favor of the upcoming task’s target dimension, resulting in speed-up of extraction of the relevant stimulus features. Although some models of task switching incorporate such attentional biasing (e.g., Meiran, 2000; see also Logan and Gordon, 2001; Meiran et al., 2008), preparation-related facilitation of task processing might, alternatively, reflect a speed-up of post-perceptual processes that transform an abstract mental code derived from the relevant perceptual attributes of the stimulus into the task-appropriate response code. Given that preparation benefits are also observed in task switching situations in which tasks do not differ regarding the relevant perceptual attributes of the stimuli (e.g., switching between classifying visually presented digits regarding their parity versus their magnitude, Schuch and Koch, 2003), assuming a post-perceptual locus of the preparation effect might be considered a more parsimonious account.

On the other hand, evidence suggesting directing of attention to a perceptual target stimulus dimension during preparation has been obtained in visual search studies. Specifically, using cross-dimension singleton search, in which participants have to detect a feature singleton that occurs randomly in one of two or more distinct perceptual dimensions, such as color or orientation, Müller H.J. et al. (2003) demonstrated that search performance benefits from advance cuing of the upcoming target’s perceptual dimension. Because the number of targets used in each perceptual dimension was limited, the cue not only indicated the perceptual dimension of the upcoming target but also constrained the set of possibly upcoming targets (e.g., to a line tilted by one of three possible degrees when the cued dimension was orientation) as well as of the upcoming S-R relation (e.g., 45° left → left key, 90° → central key, 45° right → right key), thus allowing for a non-perceptual locus of the cuing benefit. This criticism seems difficult to apply, however, to the observation of cue-based facilitation for all targets of a dimension when the cue indicated only one of them as likely to occur.

Another argument supporting preparatory adoption of attentional sets that bias processing toward the perceptual target dimension of an upcoming task relates to cuing effects observed in task switching studies in which the tasks do not differ regarding their (instructed) S-R rules. Specifically, benefits of advance task cuing have also been found when participants switched between a Stroop task (Stroop, 1935) that requires naming the print color of a color word and the complimentary task of word reading, or between responding to the global versus the local letter of a hierarchical (Navon) stimulus (Navon, 1977) with a constant letter-response assignment (e.g., H → left key, S → right key) (Hübner, 2000; Lamb et al., 2000). Inferring preparatory biasing of stimulus dimensions from these findings appears straightforward if task processing in such situations is characterized by, first, transforming the relevant perceptual attribute (e.g., red color or the letter string RED) into an abstract code which is then subjected to the same S-R translation process. However, extant models have tended to assume different, task-specific, sets of S-R translation processes even for these cases (e.g., Gilbert and Shallice, 2002), thus allowing a post-perceptual locus of the cuing benefit.

In summary, although adjustment of attentional weights given to the currently relevant and irrelevant perceptual stimulus dimensions appears to be a likely means of preparation when switching between tasks, experimental evidence that cannot alternatively be accounted for in terms of the activation of task-specific (post-perceptual) S-R translation processes is widely lacking. A possible means to control the confound of cuing the perceptual target dimension and cuing task-specific S-R rules in task switching situations is to assess task preparation when participants switch between two tasks, A and B, that are associated with different perceptual target dimensions, by means of designing another task, C (involving an unrelated S-R mapping), for which it can be argued that its execution would be differentially affected by biasing perceptual processing in favor of the target stimulus dimension of task A or of task B. Administering this *probe task* after preparation for task A versus after preparation for task B could then be informative about preparatory adjustment of processing task-specific perceptual dimensions. In the current study we applied this method to a task switching paradigm which required switching between tasks associated with selective processing of visual stimulus information presented in a smaller versus in a larger region of space (i.e., the central part of a stimulus configuration versus the whole configuration).

Selective processing of visual stimulus information presented in a particular region of space has been studied extensively under the heading of visuo-spatial attention. An often used method involves the presentation of a target stimulus at a predictable location, surrounded by task-irrelevant distractors (i.e., stimuli that do not include information necessary to solve the task and that may interfere with task performance by being associated with an incorrect response), referred to as *flankers* (i.e., flanker task, for an overview see Eriksen, 1995). Taking the *Flanker Compatibility Effect (FCE)*, that is, the performance difference between trials involving flankers associated with the same response as the target (henceforth *compatible* condition) and trials involving flankers associated with a different response than the target (henceforth *incompatible* condition), as an indicator for the degree of flanker processing, previous research demonstrated attentional focusing on the target location by demonstrating a reduction of the FCE when the spatial distance of target and flankers was increased (e.g., Eriksen and Eriksen, 1974).

Previous research using flanker tasks with variable target locations suggests that preparation time can be used to focus attention on a specific region of space. Such studies found a negative relation of flanker interference and the precision with which the location of the target was cued in advance of the presentation of the imperative stimulus (Eriksen and St. James, 1986). Consistent with these behavioral findings, an fMRI study demonstrated more widely distributed activity in visual cortex when cuing involved a larger set of possible target locations (Müller N.G. et al., 2003).

A different method of assessing the focusing of attention was introduced by LaBerge (1983). Intermixing trials of a visual search task into blocks of trials involving either a task that implied focal attention (i.e., classifying the central letter of a five-letter word) or a task associated with broader distribution of attention (i.e., classifying the meaning of a five-letter word), this author examined the spatial distribution of attention across the five locations in which the letters were presented. In the search task, a target character could occur at any of these locations with equal probability. Consistent with the assumption that differential attentional sets are adopted in the two types of task blocks, search times displayed a pronounced center-to-periphery gradient across the five locations when the search task was intermixed with trials of the focal attention task, whereas they were hardly affected by the target location when the search task was intermixed with trials of the “defocusing task”.

Intermixing a search task as a probe for the distribution of visual attention across a set of locations used for stimulus presentation in a flanker task, Wendt et al. (2012) observed a steeper search time gradient when search task trials were presented in blocks of flanker task trials that were associated with frequent compared to infrequent conflict, suggesting enhanced focusing of visual attention in response to frequent conflict evoked by the flankers. Intermixing search task trials into blocks of trials of a flanker task with asymmetrical stimuli (i.e., two identical copies of the central letter presented on one side and two instances of a different letter on the other side), Wendt et al. (2014a) obtained a steeper search time gradient when instructions asked participants to respond to the central character than to the letter presented three times (which was always identical to the central letter, yielding equivalence of the two task instructions regarding the target-response relation on each trial), suggesting that attention was focused more strongly on the location of the central letter in the former case.

Noteworthy, studies involving the probe task method to assess the attentional set associated with different tasks (LaBerge, 1983), with different frequencies of flanker conflict (Wendt et al., 2012), or with different task instructions (Wendt et al., 2014a) have so far been based on blockwise manipulations of attention, precluding conclusions about trial-to-trial adjustment of visual attention. A recent study by Longman et al. (2013), however, recorded eye movements when participants switched between responding to the photograph of a face or to a letter, superimposed on the face’s forehead. Eye fixations on regions relevant for the currently irrelevant task were more frequent in task switch than in task repetition trials, and this difference was reduced when the preparation interval was increased, suggesting both persistence and preparatory adjustment of (overt) attention.

Presenting tasks that differ regarding their demands of visuo-spatial stimulus selection and intermixing trials of a search task to probe the distribution of visual attention across a region of the visual field seems a promising means to assess, selectively, persistence and preparation of task-specific sets of visual attention, as a particular component of the task-set. In the current study, we applied this methodological approach. Experiment 1 involved a conceptual replication of previous studies that found differential search time patterns consistent with assumptions made regarding attentional sets adopted for different tasks or other context conditions, presented in the majority of trials (LaBerge, 1983; Wendt et al., 2012, 2014a). More specifically, two tasks afforded by the same set of stimuli were alternated between blocks of trials. One of the tasks required responding to the identity of a centrally presented target character and ignoring adjacent stimulus characters that could be identical to or different from the target (i.e., an Eriksen flanker task). By contrast, the other task required judging whether all characters were the same or not. In both task blocks, we intermixed trials of a search task involving a target stimulus that could occur in any of the three possible character locations. Because the context task was kept constant for a block of trials search time patterns may reveal attentional sets adopted in a sustained manner and are not informative regarding (trial-to-trial) dynamics of attentional adjustment in response to (anticipated) changing attentional demands. In Experiment 2, we pursued this issue by examining search time patterns as a function of preparation for each of the other two tasks as well as a function of previous execution thereof.

## Experiment 1

Vertically arranged strings of three letters served as stimuli for two different tasks. Only the letters H and S were used, and the top and bottom position always involved the same letter. To manipulate stimulus selection demands, an Eriksen flanker task, in which participants identified the centrally presented letter, was contrasted with a Same/Different task, in which participants judged whether all three letters were identical or not. To probe the distribution of visual attention across the three locations at which letters occurred, a visual search task was used. In this task, three digits were presented in the same locations as the letters in the letter tasks. One of these locations, randomly chosen on each trial, contained one of two possible target digits, which participants were instructed to identify. We expected to replicate and extend previous findings of a steeper center-to-periphery gradient of search times in blocks of trials in which the context task was assumed to be associated with stronger attentional focusing on the central location (LaBerge, 1983; Wendt et al., 2012, 2014a).

### Method

#### Participants

Six female and 14 male students of the Helmut Schmidt University/University of the Federal Armed Forces Hamburg, ranging in age from 21 to 29 years, participated in a single-session experiment in exchange for partial fulfillment of course requirements.

#### Apparatus and Stimuli

The experiment took place in a silent, dimly lit room with a 19-inch. LCD monitor with a refresh rate of 60 Hz. Subjects sat approximately 80 cm away from the monitor. Responses were given by pressing one of two response keys which were mounted on an external rectangular keyboard (10 cm × 18 cm). The response keys extended 1.0 cm × 1.0 cm and were separated by 8.0 cm (parallel to the keyboard’s long axis). Participants pressed the response keys with the index or middle fingers of their left and right hands (hands uncrossed).

The stimuli were presented in white on a dark gray background, inside a thin white rectangular frame (96 mm × 102 mm) in the center of the screen. In both the letter tasks and in the search task the stimuli involved three-element-strings, presented in vertical format. In both letter tasks a central capital letter (*H* or *S*) was flanked by either two copies of the same or the alternative letter, forming stimuli with same (*HHH*, *SSS*) or with different letters (*SHS*, *HSH*). Search task stimuli were made up of three different digits drawn from the set of 0–9. Letters and digits extended from 5 to 10 mm horizontally, depending on the precise character, and 12 mm vertically. A three-element-string subtended approximately 0.72° horizontally and 3.8° vertically. In the Eriksen task, responses to the target letter *H* and *S* were mapped to the left and right response key, respectively. In the Same/Different task homogeneous and heterogeneous letter strings were assigned the left and the right response key, respectively. In the search task, the target digits *3* and *7* were mapped to the left and right response key, respectively.

#### Procedure

Participants were first administered three practice blocks, involving 40 trials each. The first practice block comprised only trials of the search task. The second practice block comprised trials of the Eriksen task and trials of the search task. The third practice block comprised trials of the Same/Different task and trials of the search task. All constraints of task and stimulus selection were identical to the constraints of the experimental blocks. Subsequently, 12 experimental blocks, of 99 trials each, were started. Blocks involving the Eriksen task and blocks involving the Same/Different task were presented alternately, and the order of presentation was counterbalanced across participants. Search task trials were never presented on consecutive trials. Following a letter task trial (i.e., Eriksen task or Same/Different task, depending on the current block), the probabilities of a search task trial or of another letter task trial were 50%, each. In the letter tasks, the central stimulus element and the peripherally presented stimulus elements (which always matched) were chosen randomly on each trial, yielding 50% probabilities for both a homogenous and a heterogeneous letter string. In the search task, the target digit (i.e., *3* or 7) and the target location (i.e., top, central, and bottom) were randomly chosen. Two additional digits (differing from the possible target digits and from each other) were randomly chosen for the remaining two locations. Participants were instructed to respond as fast as possible while avoiding errors.

After each block, participants received written feedback about their average RT and error rate of the block and were informed about the letter task of the upcoming block. They were given the opportunity for a self-timed pause. Letter and digit stimuli were presented for 150 ms. In case of a correct response the next stimulus appeared after 1300 ms. After an incorrect response the word “falsch” (“wrong”) was presented at the bottom of the stimulus frame for 800 ms. Again, the next stimulus appeared after 1,300 ms. An experimental session took from 50 to 65 min.

### Results

The first three trials of each block were considered “warm-up” trials and not analyzed. In addition, data from trials following an erroneous response as well as data from trials associated with RTs deviating more than 2.5 standard deviations from the mean RT of each experimental condition per participant were discarded from the statistical analyses.

Although only performance in the search task was of primary interest with regard to the purpose of our study, we also analyzed the response data in the two letter tasks. Specifically, RTs and error percentages were broken down to the factors Task (Eriksen, Same/Different), Homogeneity/Heterogeneity of the letter string, and Congruency between tasks (i.e., whether a given letter string required the same or different responses in the two tasks [i.e., congruent and incongruent, respectively]). (We chose the labels “homogeneous” and “heterogeneous” to refer to the letter strings, rather than the common labels “flanker-compatible” and “flanker-incompatible” because the latter would not seem appropriate for the Same/Different Task, in which the letters presented in peripheral locations do not act as [compatible or incompatible] distractors). Descriptive statistics are presented in **Table 1**.

**Table 1:** Mean reaction times in ms and error percentages (in parantheses) of the letter task trials in Experiment 1 as a function of Task (Eriksen, Same/Different), Stimulus Type (homogenous, heterogeneous), and Response Congruency between tasks.

	Eriksen		Same/Different	
	Congruent	Incongruent	Congruent	Incongruent
Homogenous	489 (4.1)	494 (4.2)	491 (4.4)	501 (6.1)
Heterogeneous	509 (3.9)	519 (4.9)	536 (3.7)	538 (3.7)

**FIGURE 1:**
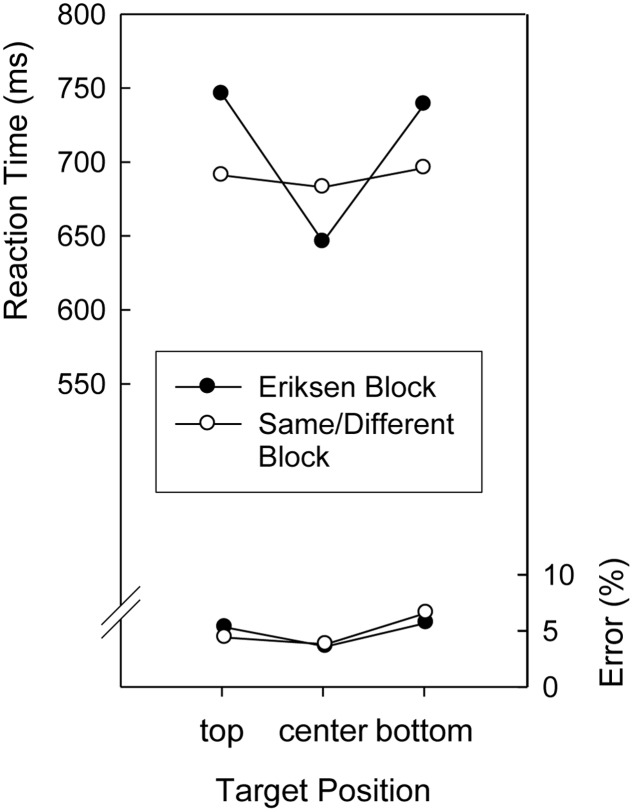
**Mean reaction times and error percentages in the search task of Experiment 1 as a function of target position and block (Eriksen, Same/Different)**.

Mean RTs and error percentages in the search task are displayed in **Figure 1**. To analyze performance in the search task, an Analysis of Variance (ANOVA) was conducted on the mean RTs with repeated measures on the factors Target Position (top, center, and bottom) and Context Letter Task (Eriksen, Same/Different). Both the main effects of Context Letter Task and Target Position as well as the two-way-interaction were significant, *F*(1,19) = 12.3, *p* < 0.01, *MSE* = 1,023.1, *F*(2,38) = 18.5, *p* < 0.01, *MSE* = 2,082.0, and *F*(2,38) = 18.7, *p* < 0.01, *MSE* = 1,327.1, respectively. As can be seen in **Figure 1**, a pronounced center-to-periphery gradient of search times occurred in the Eriksen Task blocks but not in the Same/Different Task blocks. RTs were shortest for centrally presented targets in the Eriksen Task blocks, longest for targets presented in non-central locations in the Eriksen Task blocks, and intermediate for all locations in the Same/Different Task blocks. A corresponding ANOVA on the mean error proportions yielded only a significant main effect of Target Position, *F*(2,38) = 5.77, *p* < 0.01, *MSE* = 0.1298, indicating that fewer errors were made when the target digit was presented in the central position than when it was presented in peripheral locations (see **Figure 1**)^1^.

### Discussion

The results of Experiment 1 extended previous findings that search time patterns in intermixed trials of a visual search task can be affected by the stimulus selection demands or by attention-relevant stimulus-response contingencies of a context task (e.g., LaBerge, 1983; LaBerge and Brown, 1989; Wendt et al., 2012, 2014a). Specifically, as expected on the assumption that participants adopt a more focused set of visual attention in blocks of trials including predominantly Eriksen flanker task trials than in blocks of trials including predominantly trials of a task requiring a homogeneous/heterogeneous judgment regarding a target-flanker ensemble, the RT pattern in intermixed trials of a visual search task displayed a more pronounced center-to-periphery gradient in the former case. Although the precise processes underlying the different search time patterns can be debated (see “General Discussion”), we note that it seems difficult to ascribe this finding to other differences between the two letter tasks than their “spatial target regions,” such as the task-specific matching operations (i.e., comparing a current stimulus with a memory representation versus with another currently presented stimulus) or the task-specific S-R translation rules. Thus the results of Experiment 1 suggest that the search task employed provides a useful means to assess the set of visual attention associated with a different task that comprises a different set of stimuli.

Because the search task was intermixed into blocks of trials associated with either the Eriksen flanker task or the Same/Different task, the difference in search time patterns may reflect a sustained form of adoption of task-specific sets of visual attention, kept more or less constant throughout a block of trials. As an alternative, it may result from persistence of a set, used on the direct predecessor trial, or, given that the search task always occurred with lower likelihood than the letter task of the block, from preparatory re-adoption of a rapidly decayed set during the pre-target interval.

## Experiment 2

In Experiment 2, trial-to-trial persistence and preparation of task-specific attentional sets were investigated by intermixing the Eriksen task and the Same/Different task in the same block of trials and presenting cues that indicated the upcoming task in advance of the imperative stimulus. To assess task-specificity of visual attention, again, search task trials occurred unpredictably, that is, after a letter task cue. Assuming preparatory adoption of the attentional set associated with the cued task we expected a more pronounced center-to-periphery gradient of search times after a cue that indicated the Eriksen task than after a cue that indicated the Same/Different task. Similarly, assuming persistence of the attentional set associated with the letter task executed on the preceding trial we expected a more pronounced center-to-periphery gradient of search times after an Eriksen task trial than after a Same/Different task trial.

To control for possible “exogenous” cuing effects (i.e., focusing of attention to the region covered by the cue), the experiment was run in two versions. The procedure of these versions differed only regarding the type of cues used: Version 1 involved written words, presented in the center of the screen, whereas version 2 involved three vertically arranged disks, displayed in a task-specific color, that covered the whole area of a three-letter/digit string.

### Method

#### Participants

Forty students of the Helmut Schmidt University/University of the Federal Armed Forces Hamburg participated in a single-session experiment in exchange for partial fulfillment of course requirements. None of them had participated in Experiment 1. The word cue version (i.e., Version 1) included 9 female and 11 male participants, ranging in age from 20 to 28 years. The dots cue version (i.e., Version 2) included 3 female and 17 male participants, ranging in age from 20 to 29 years.

#### Apparatus and Stimuli

Identical to Experiment 1, with the exception that each stimulus of the letter tasks and of the search task was preceded by the presentation of a task cue. In Version 1 the cues were the words “Mitte” (“center”), indicating the Eriksen task, and “Gesamt” (“entire”), indicating the Same/Different task. The word “Mitte” extended 25 mm horizontally and 8 mm vertically (about 1.8° × 0.6° of visual angle), and the word “Gesamt” extended 32 mm horizontally and 8 mm vertically (about 2.3° × 0.6°of visual angle). The cues were presented in the center of the screen in white color. In Version 2, the cues were three vertically arranged colored disks presented in the same positions as the letter or digit stimuli. They measured 15 mm in diameter each (around 1.1°, all three 1.1° × 3.8°). The disks were either yellow or cyan. Balanced across participants yellow disks indicated the Eriksen task and cyan-colored disks indicated the Same/Different task or vice versa.

#### Procedure

The procedure of Experiment 2 was identical to the procedure of Experiment 1 with the following exceptions. First, each imperative stimulus was preceded by a task cue. The cue was presented 1,300 ms after a response was made to the preceding trial’s stimulus and remained on the screen for 800 ms, directly followed by the imperative stimulus. Second, the practice phase involved four blocks of trials. A first practice block comprised only Eriksen task trials, a second practice block comprised only Same/Different task trials, and a third practice block comprised only search task trials. These blocks included 20 trials each. Each trial started with the presentation of a task cue. (For the letter task blocks, the cue was redundant. In the search task block, the cue was chosen randomly from the two letter task cues on each trial). A final practice block of 40 trials involved the presentation of all three tasks with the same constraints of task and stimulus selection that were realized in the following 12 experimental blocks (that comprised 99 trials each). Third, all experimental blocks were structurally identical. They involved the presentation of all three tasks, preceded on each trial by a task cue. Whereas letter task trials were always validly cued (i.e., preceded by their corresponding task cue), on search task trials the cue was chosen randomly from the two possible letter task cues. After a search task trial, the Eriksen task and the Same/Different task occurred with a probability of 50% each. (The search task was never presented on consecutive trials). After a letter task trial the probability for each of the letter tasks was 4/11 and the probability for the search task was 3/11. An experimental session took from 55 to 65 min.

### Results

The same routines for data exclusion were applied as in Experiment 1. Descriptive values of the performance in the letter tasks are displayed in **Table 2**.

**Table 2:** Mean reaction times in ms and error percentages (in parantheses) of the letter task trials in Experiment 2 as a function of Version (1/word cues, 2/color cues), Task (Eriksen, Same/Different), Stimulus Type (homogenous, heterogeneous), and Response Congruency between tasks.

	Version 1 (word cues)				Version 2 (color cues)			
	Eriksen		Same/Different		Eriksen		Same/Different	
	Cong	Incong	Cong	Incong	Cong	Incong	Cong	Incong
Hom	618 (4.4)	615 (14.0)	554 (4.3)	614 (12.7)	686 (2.2)	748 (10.0)	640 (2.3)	712 (7.9)
Het	634 (6.2)	648 (9.9)	649 (5.6)	687 (12.4)	740 (3.8)	741 (9.3)	703 (3.4)	758 (9.8)

*Cong, congruent; incong, incongruent; hom, homogenous; het, heterogeneous.*

**FIGURE 2:**
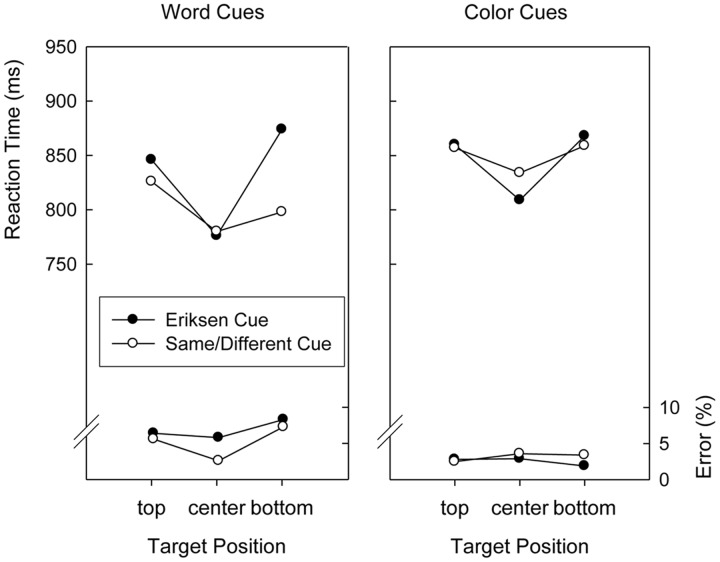
**Mean reaction times and error percentages in the search task of Experiment 2 as a function of version (word cues, color cues), cued task, and target position**.

Performance in the search task was analyzed by conducting ANOVAs with repeated measures on the factors Version (word cues, color cues), Cued Task (Eriksen, Same/Different), Preceding Task (Eriksen, Same/Different), and Target Position (top, center, and bottom), on the mean RTs and on the mean error proportions. Regarding RTs, a main effect of Target Position, *F*(2,76) = 21.3, *p* < 0.01, *MSE* = 5,908.0, indicated a clear center-to-periphery gradient of search times that was modulated by Cued Task, yielding a significant two-way interaction, *F*(2,76) = 3.9, *p* < 0.03, *MSE* = 8,558.8. **Figure 2** displays the pattern of search times for the cuing conditions, separately for the two versions of the experiment. A planned comparison that contrasted quadratic trends across the three target positions demonstrated that the center-to-periphery gradient of search times was more pronounced after Eriksen task cues than after Same/Different task cues, *F*(1,38) = 16.2, *p* < 0.01, *MSE* = 2,852.1. This was not modulated by Version, as demonstrated by another planned comparison, *F*(1,38) < 1.

As can be seen in **Figure 2**, responses after Eriksen task cues were slower than responses after Same/Different task cues in Version 1 and slightly faster in Version 2, resulting in both a significant main effect of Cued Task, *F*(1,38) = 6.1, *p* < 0.02, *MSE* = 3,321.5, as well as a significant two-way interaction with Version, *F*(1,38) = 10.9, *p* < 0.01, *MSE* = 3,321.5.

**FIGURE 3:**
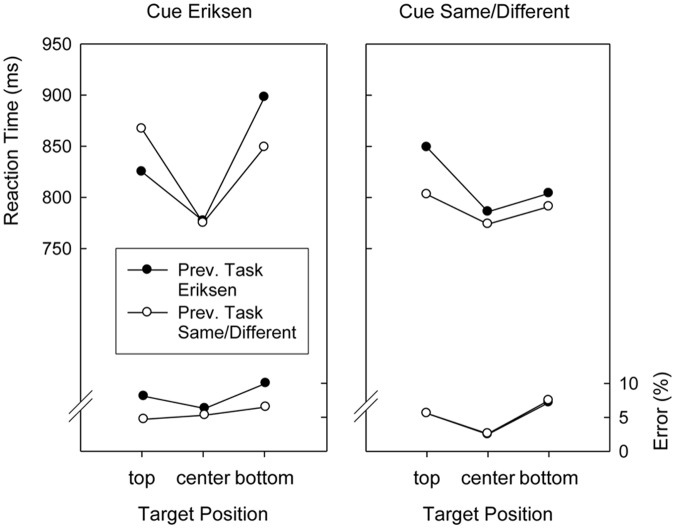
**Mean reaction times and error percentages in the search task of Version 1 (word cues) of Experiment 2 as a function of cued task, previous task, and target position**.

**FIGURE 4:**
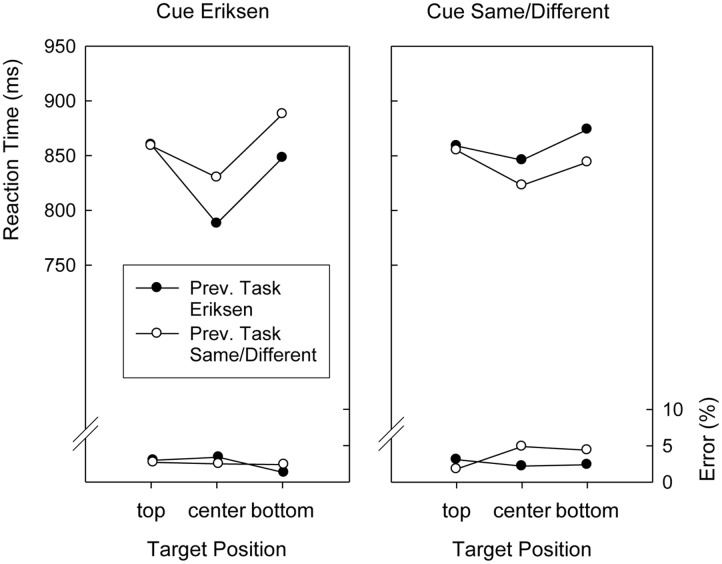
**Mean reaction times and error percentages in the search task of Version 2 (color cues) of Experiment 2 as a function of cued task, previous task, and target position**.

Although Target Position did not interact with Preceding Task, *F*(2,76) < 1, the two-way interaction involving Cued Task and Preceding Task was significant, *F*(1,38) = 14.8, *p* < 0.01, *MSE* = 2,215.8. As can be seen in **Figures 3, 4**, this was because responding after an Eriksen task cue was faster after Eriksen task trials than after Same/Different Task trials (833 ms versus 845 ms), whereas responding after a Same/Different task cue was faster after Same/Different Task trials than after Eriksen task trials (815 ms versus 836 ms). Finally, all factors entered into a complicated four-way interaction, *F*(2,76) = 3.4, *p* < 0.04, *MSE* = 7,099.7, displayed in **Figures 3, 4**.

A corresponding ANOVA on the mean error proportions yielded a significant main effect of Version, *F*(1,38) = 6.4, *p* < 0.02, *MSE* = 0.01861, indicating that overall fewer errors were made with color cues (i.e., Version 2, 2.8%) than with word cues (i.e., Version 1, 6.0%). This was modulated by significant two-way interactions with Cued Task and Preceding Task, *F*(1,38) = 4.4, *p* < 0.05, *MSE* = 0.00344, and *F*(1,38) = 5.5, *p* < 0.03, *MSE* = 0.001728, respectively. Responses were more error-prone after Eriksen task cues than after Same/Different task cues in Version 1 (6.8% versus 5.2%), and this was slightly reversed in Version 2 (3.1% versus 2.5%). Also, responses were more error-prone after Eriksen task trials than after Same/Different task trials in Version 1 (6.6% versus 5.4%), and this was slightly reversed in Version 2 (3.1% versus 2.6%).

The main effect of Target Position was marginally significant, *F*(2,76) = 2.5, *p* = 0.08, *MSE* = 0.00352, and only modulated by Version, *F*(2,76) = 5.2, *p* < 0.01, *MSE* = 0.00352. As can be seen in **Figure 2**, a center-to-periphery gradient of response accuracy occurred when word cues were used but not when color cues were used.

Finally, the two-way interaction of Cued Task and Preceding Task was marginally significant, *F*(1,38) = 3.4, *p* = 0.07, *MSE* = 0.00342, because responding in trials involving an Eriksen task cue was more error-prone after Eriksen task trials than after Same/Different Task trials (5.4% versus 0.4%), whereas responding in trials involving a Same/Different task cue was more error-prone after Same/Different Task trials than after Eriksen task trials (4.5% versus 3.8%).

### Discussion

The most important result of Experiment 2 was that the pattern of search times was affected by the to-be-expected task. Specifically, a more pronounced center-to-periphery gradient occurred after a cue indicating an Eriksen flanker task, that required the identification of the central element of a three-letter string, than after a cue indicating a Same/Different task, in which homogeneity/heterogeneity of the whole letter string had to be evaluated. This finding was expected on the assumption that participants would adjust their ability to process visual stimulus information to the characteristics of the anticipated task, focusing attention to a particular location or distributing it over a wider area.

The pattern of search task performance was also affected by the type of cue used although this effect was only significant in the error analysis. Specifically, we observed an advantage for targets presented in the central location in Version 1, but not in Version 2, in which color cues were used. This finding may be explained in terms of exogenous cuing of attention by the spatial position and extension of the cue. Unlike the color cues, that covered the area of the complete three-letter/digit strings, the words used as cues were presented in the horizontal midline of the screen and corresponded in height to a single digit of the search task. Cue type-induced focusing of attention could not be corroborated, however, in an analysis of the performance patterns in the letter tasks. As can be seen in **Table 2**, there was neither a relative advantage of the Eriksen task in Version 1 and of the Same/Different task in Version 2, nor a larger FCE (homogenous versus heterogeneous stimuli) in Version 2 than in Version 1. Importantly, irrespective of all possible differences in exogenous cuing of attention, the modulation of the search time gradient by the indicated task was found with both types of cues used in the experiment.

Contrasting with the cuing effect, the pattern of search task performance across the three target locations was unaffected by the type of task executed on the directly preceding trial. The experiment thus yielded no support for persistence of task-specific sets of visual attention into a subsequent trial, at least if the following trial involves a task associated with a different set of visual attention and the possibility to prepare for it.

Search task performance was, however, overall faster and (marginally significantly) more error-prone after a cue indicating a task repetition than after a cue indicating a task switch. This finding suggests that participants adjusted their response strategy to the expected task sequence (in addition to adjusting their set of visual attention to the expected task). This suggestion is consistent with the results of modeling work. Specifically, Schmitz and Voss (2012, see also Karayanidis et al., 2009), applying a diffusion model to a standard task switching situation, inferred a lower degree of response caution in (prepared) task repetition trials than in task switch trials.

## General Discussion

Intermixing trials of a probe task, designed to be sensitive to selected aspects of task-specific S-R processing, seems an efficient method to investigate persistence and preparation of specific components of task-sets. The current study aimed primarily at pursuing the set of visual attention but also yielded preliminary evidence regarding response caution. Consistent with previous results of steeper search time gradients when context conditions were arguably associated with stronger focusing of attention, Experiment 1 yielded a corresponding search time pattern when the search task was intermixed into blocks of trials of the Eriksen flanker task versus into blocks of trials of the Same/Different task, suggesting task-specific focusing or defocusing of visuo-spatial attention. The blockwise manipulation, however, precludes interpreting these results in terms of dynamic trial-to-trial adjustment.

Experiment 2 demonstrated preparation-based adoption of task-specific sets of visual attention by yielding differentially steep search time gradients after (invalid) cuing of the two letter tasks. By contrast there was no indication of a difference in search time gradients as a function of the type of the directly preceding letter task. Regarding the letter tasks, the situation created in Experiment 2 resembles typical task switching studies in which the same stimuli are administered under varying S-R mappings that have to be applied to perceptually different stimulus dimensions, such as shape and color. Although task-specific perceptual biasing during preparation has been assumed in some models of task switching (e.g., Meiran, 2000) we know of no evidence for this assertion that is comparable to the results of Experiment 2 of the current study.

Further research is needed to clarify several questions left open by the current findings. First, the lack of an influence of the attentional demands of the preceding task on the search time pattern deserves further analysis. At the current stage we can only speculate whether this reflects passive decay or inhibition of a previous attentional set or overwriting by preparation for the upcoming one. Manipulations of the length of the inter-trial interval and of the certainty regarding the identity of the upcoming task may be helpful to decide among these possibilities. Second, although the preparation effect found in the current study seems consistent with an early, sensory-perceptual locus, it may also be brought about by re-adjustment of processing weights assigned to stimulus information extracted from central and peripheral locations during a later, post-perceptual processing phase. Distinguishing between these possibilities on the basis of purely behavioral findings may be difficult. Complimentary analysis of physiological measures reflecting early processing stages of stimuli presented in the different locations seem a viable option to shed light on this issue (see, Wendt et al., 2014b; Jost et al., 2017, for application of a similar method concerning the question of adjustment of processing of distractors presented in advance of the target). Third, as noted in the “Introduction,” Longman et al. (2013) observed an effect of preparation on eye fixations in regions of relevance for the two tasks between which participants switched, suggesting that overt attentional selection is prepared when switching between tasks. Although situations in which stimulus information relevant for the two tasks is presented in different locations seem particularly likely (and actually necessary, given a critical distance between these locations is exceeded) to be associated with task-specific sets of overt attentional selection (i.e., different fixation points), presenting critical information in a smaller region of space in one task than in the other may also invoke functional differences in eye movements or fixation patterns. Because we did not control eye movements, it can thus not be dismissed that the preparation effect was brought about by overt rather than covert attentional focusing.

Potentially limiting the scope of our findings it should be pointed out that switching between tasks which require differential degrees of focusing of visuo-spatial attention, as done in the current study, seems particularly well-suited for investigations based on intermixing a probe task because clear predictions can be made regarding RT patterns (i.e., steeper search time gradient in the context of a task that requires stronger focusing). Generalization of such findings to more typical task switching situations, such as switching between color and shape classifications, seems premature, however. Given the promising results of the current study, however, substantial progress regarding the questions of persistence and preparation of processing task-specific perceptual dimensions, in general, may be made if appropriate probe tasks can be developed for other kinds of stimulus dimensions than the size of the spatial region containing critical stimulus information. An obvious problem inherent in this approach relates to the possibility of changed task processing strategies resulting from intermixing probe task trials. For instance, regarding the current study, administering a proportion of trials in which peripherally presented stimuli bear task relevance may lead participants to adopt a less focused strategy in the Eriksen task as they would do in single-task Eriksen blocks. Constituting an example of a very reactive measurement, progress obtained with more sophisticated probe task procedures should thus depend on a more profound understanding of the adjustment of task processing strategies to contextual factors.

Although we had no *a priori* hypothesis regarding preparatory adjustment of response strategies, the speed-accuracy trade-off found in search task trials that were cued as (letter) task repetitions versus switches provides striking evidence for the notion of a lowering of the response criterion in anticipation of a task repetition, derived from modeling work (Karayanidis et al., 2009; Schmitz and Voss, 2012). Intriguingly, evidence for this suggestion in the form of a speed-accuracy trade-off between well prepared task repetitions and switches seems widely missing. This would not seem surprising given that prepared task repetitions should be particularly easy to perform, thus providing little room for erroneous responding even if response caution is reduced. The probe task method used in the current study might be advantageous in this regard because it compares performance after preparation for a task repetition and for a task switch in an unexpected task which should not benefit from the facilitation one would expect to see in the prepared task repetition, thus increasing error likelihood.

From a methodological point of view, intermixing probe task trials may thus be useful to identify several different task-set components that are affected by preparation. The current study provides clear evidence for preparatory adoption of task-specific sets of visual attention as well as a corroboration of suggestions of task sequence-specific preparation of the response strategy.

## Ethics Statement

All procedures performed in studies involving human participants were in accordance with the ethical standards of the institutional and/or national research committee and with the 1964 Helsinki declaration and its later amendments or comparable ethical standards. Informed consent was obtained from all individual participants included in the study. Ethical review and approval was not required for this study in accordance with the national and institutional requirements.

## Author Contributions

MW, AL-R, and TJ planned the experiments. AL-R programmed the experimental software. SK collected the data. MW and AL-R analyzed the data. All authors wrote the article.

## Conflict of Interest Statement

The authors declare that the research was conducted in the absence of any commercial or financial relationships that could be construed as a potential conflict of interest.

## Footnotes

1 To examine possible effects from the search task on processing of the letter tasks, we conducted an additional analysis, based only on the RT data from letter task trials preceded by a search task trial, including the factors Task (Eriksen, Same/Different), Homogeneity/Heterogeneity of the letter string, Congruency between tasks, and Target position on the preceding trial (central, peripheral). This analysis yielded a significant three-way interaction involving Task, Homogeneity/Heterogeneity of the letter string, and Target position on the preceding trial, *F*(1,19) = 8.2, *p* < 0.02, *MSE* = 4161.8, demonstrating that responding to a heterogeneous letter string in the flanker task was slowed by a preceding search task target presented at a peripheral location whereas no such slowing occurred for homogeneous letter strings. A similar result was observed by Wendt et al. (2012). Consistent with the notion that the slowing observed for heterogeneous letter strings reflected an increase in response competition due to higher deployment of attention to a flanker stimulus (i.e., persistence of attentional orienting of the preceding search task trial), no such slowing occurred in the Same/Different Task for either type of letter string.
